# Geometrical Effects on Ultrasonic Al Bump Direct Bonding for Microsystem Integration: Simulation and Experiments

**DOI:** 10.3390/mi12070750

**Published:** 2021-06-26

**Authors:** Jun-Hao Lee, Pin-Kuan Li, Hai-Wen Hung, Wallace Chuang, Eckart Schellkes, Kiyokazu Yasuda, Jenn-Ming Song

**Affiliations:** 1 Department of Materials Science and Engineering, National Chung Hsing University, Taichung 402, Taiwan; r840722@dragon.nchu.edu.tw (J.-H.L.); klausli@spil.com.tw (P.-K.L.); hung523@yahoo.com.tw (H.-W.H.); 2 Automotive Electronics Department, Robert Bosch Taiwan Co., Ltd., Taipei 104, Taiwan; Wallace.Chuang@tw.bosch.com (W.C.); Eckart.Schellkes@tw.bosch.com (E.S.); 3 Division of Materials and Manufacturing Science, Graduate School of Engineering, Osaka University, Osaka 565-0871, Japan; yasuda@mapse.eng.osaka-u.ac.jp; 4 Research Center for Sustainable Energy and Nanotechnology, National Chung Hsing University, Taichung 402, Taiwan; 5 Innovation and Development Center of Sustainable Agriculture, National Chung Hsing University, Taichung 402, Taiwan

**Keywords:** finite element analysis, ultrasonic bonding, metal direct bonding, microsystem integration

## Abstract

This study employed finite element analysis to simulate ultrasonic metal bump direct bonding. The stress distribution on bonding interfaces in metal bump arrays made of Al, Cu, and Ni/Pd/Au was simulated by adjusting geometrical parameters of the bumps, including the shape, size, and height; the bonding was performed with ultrasonic vibration with a frequency of 35 kHz under a force of 200 N, temperature of 200 °C, and duration of 5 s. The simulation results revealed that the maximum stress of square bumps was greater than that of round bumps. The maximum stress of little square bumps was at least 15% greater than those of little round bumps and big round bumps. An experimental demonstration was performed in which bumps were created on Si chips through Al sputtering and lithography processes. Subtractive lithography etching was the only effective process for the bonding of bumps, and Ar plasma treatment magnified the joint strength. The actual joint shear strength was positively proportional to the simulated maximum stress. Specifically, the shear strength reached 44.6 MPa in the case of ultrasonic bonding for the little Al square bumps.

## 1. Introduction

Recent research has promoted the application of ultrasonic bonding, which is conventionally used in the bonding of metal/plastic or plastic/plastic, to metal direct bonding. Ultrasonic bonding was recently developed for hermetic sealing in microelectromechanical system (MEMS) packaging. Since 2002, ultrasonic bonding has been used in homogenous and heterogenous metal bonding, including Al/Al, In/Au, and Au/Al [1,2]. Since 2007, studies on the application of ultrasonic bonding to tinned Cu columns have emerged [3,4,5], and the first report of successful direct Cu bump bonding appeared in 2013. At present, metal direct bonding, including Cu rim sealing in sensors or flip chip bonding of Au or Cu bumps, is under development in the packaging processes of microelectromechanical systems [6,7,8]. All these progresses are due to the advantages of ultrasonic bonding, i.e., the extremely short bonding time (in seconds or less than 1 s) and the operability at room temperature in ambience.

In ultrasonic bonding [9], fast horizontal vibration is generated using ultrasound to bond two metal surfaces through rapid friction at the bonding interface. The friction damages the surface oxidation layers, removes impurities, and generates a high thermal energy, thereby drastically reducing the bonding time. This method does not require the use of a flux. The short bonding time generates little byproducts (e.g., intermetallic compounds) and therefore allows the resulting component to maintain its favorable electrical properties. Despite advantages such as short bonding time, high bonding strength, high electrical conductivity, and low processing temperature, ultrasonic bonding is less effective when the bonding surface is large.

ANSYS (v.19.2, 2018, Ansys, Inc., Canonsburg, PA, USA) is a program capable of simulating the stress of a material subject to force. Regarding the use of ANSYS in the simulation of ultrasonic bonding, Wang et al. [10] simulated the stress and strain generated by the bonding of composite polymer materials and Al, and compared the simulated joint shear strength with the value obtained in an experimental demonstration. Arai et al. [11] simulated horizontal and vertical deformation in bumps subject to ultrasonic bonding and suggested that high deformation is associated with high bonding strength. Sasaki et al. [12] simulated the effect of different welding heads on ultrasonic bonding and reported a positive correlation between bonding strength and the depth of stress distribution. In ultrasonic bonding, friction exerts a substantial effect on the stress and strain distributions. Increasing the frequency of friction increases the equivalent strain of the corner surface. This indicates that when the equivalent strain is highly concentrated at the corner of a workpiece, the resulting deformation becomes less even [13]. Myung et al. [14] considered thermal cycle parameters in finite element analysis (FEA) of Cu/Cu bonds, the formation of cracks could be predicted.

This study aimed to improve the feasibility of ultrasonic bonding in microsystem integration and employ metal bump direct bonding as an alternative to conventional polymer and solder joining techniques, such as die attachment, flip chip, and ball grid array packaging. As for flip chip, the arrays of bumps with the functions of mechanical support and electrical and thermal conductance usually consist of solders. Studs of gold, copper, silver, and their alloys have also been applied [15,16,17,18,19]. In such cases, intermetallic compounds usually form at the interface with Al pads and likely lead to reliability problems, especially when subject to a harsh environment. Al bump arrays made from bonding wires were recently developed to join with Al pads for high temperature applications such as SiC power devices [20]. This joint structure is free from the formation of interfacial intermetallic compounds and the subsequent problems. To optimize the ultrasonic Al bump bonding process, we attempted to conduct experiments on and simulate the use of ultrasonic bonding to integrate Al bump arrays with Al thin film. In addition to using ANSYS to simulate the stress distribution of bumps subject to force, this study includes an experimental demonstration to determine the effects of bump shape and size on simulated maximum stress and actual joint strength.

## 2. Experimental Procedures

### 2.1. Finite Element Analysis

**Figure 1:**
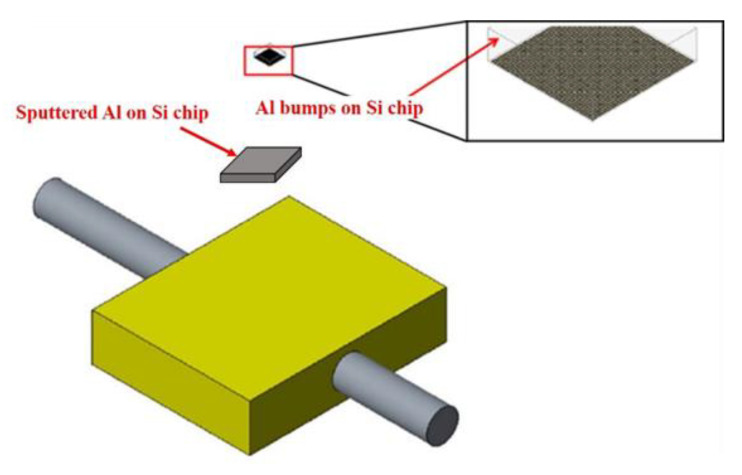
Simulation setup for ultrasonic bonding in this study.

**Figure 2:**
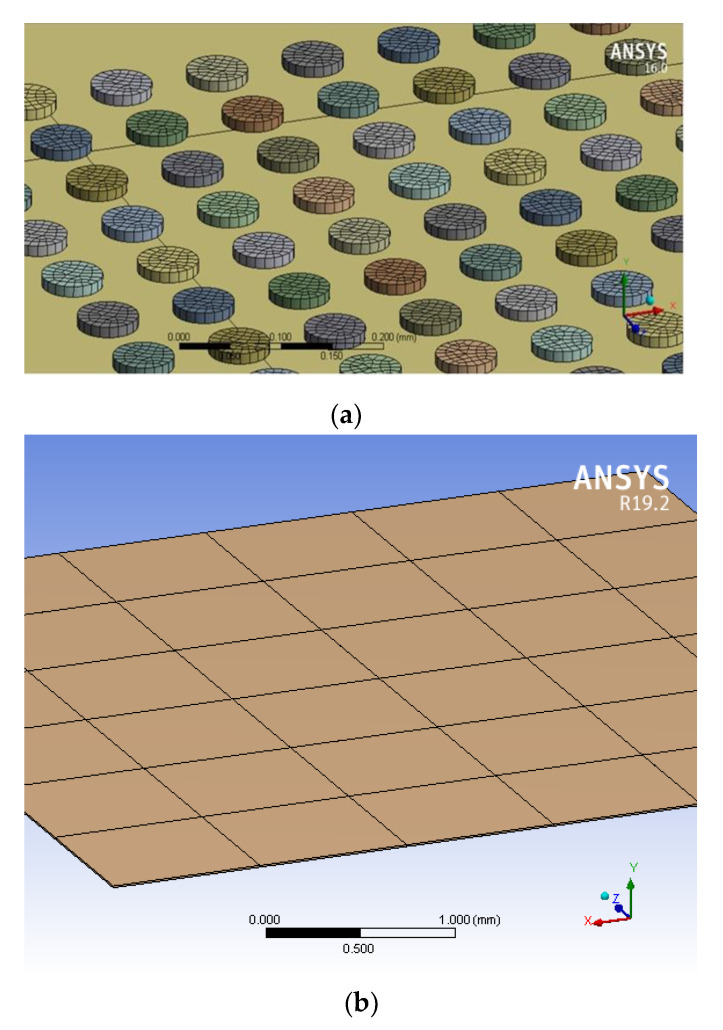
Meshing for simulation: (**a**) 10 μm-thick big round bumps (BRB), (**b**) 1 μm-thick Pd for Ni/Pd/Au.

To explore the effect of bump shape and size on the effectiveness of ultrasonic bonding, FEA was performed to simulate the stress exerted on the bump surface during the bonding process. Figure 1 presents the setup for simulation. First, 3D software was used to create a model and generate three types of bumps, namely little square bumps (LSB), little round bumps (LRB), and big round bumps (BRB). The bumps were stacked on a chip coated with metal thin films, and the workpiece was placed on a platform with a heating rod. Model meshing was performed based on the bump size. Next, the static structural model was selected in ANSYS. The steady-state thermal model was then selected and linked with the static structural model to configure the properties of the materials, namely Al, Cu, and Ni/Pd/Au. Owing to limitations in computation time and capability, the total bump height was set as 5 μm and 10 μm, and the thickness of Pd and Au was set as 1 μm. With respect to the meshing of samples, the adaptive sizing mode was used. Minimum edge length was the bump height, i.e., 5 μm for 5 μm-thick bumps, and 10 μm for 10 μm-thick bumps. As in the example given in Figure 2a, the 5 μm and 10 μm-thick bumps were all divided into 44 cells. As for the Pd or Au with the thickness of 1 μm (Figure 2b), the minimum edge length was 1 μm and meshing cell number was 36.

All material parameters were set according to theoretical values (Table 1); for example, the density, Young’s modulus, and Poisson’s ratio of Al were 2.7 g/cm^3^, 70 GPa, and 0.35, respectively. The configured model was input into ANSYS to determine the model materials. Figure 2 illustrates the model meshing. The boundary condition, set using the fixed support model, of force was determined as the upper surface of the die surface, with an input force of 200 N. Vibration displacement time and distance were input in the bump movement. For the steady-state thermal model setting, the heating rod temperature was set as 200 °C. Notably, frictional displacement was considered in the simulation to accurately reflect the actual process of ultrasonic bonding. This was achieved by configuring the material friction coefficient (Table 1), frictional displacement (6 μm), and frictional frequency (35 kHz). Finally, the equivalent stress was input to solve the final stress value.

### 2.2. Preparation of Al Bumps

**Figure 3:**
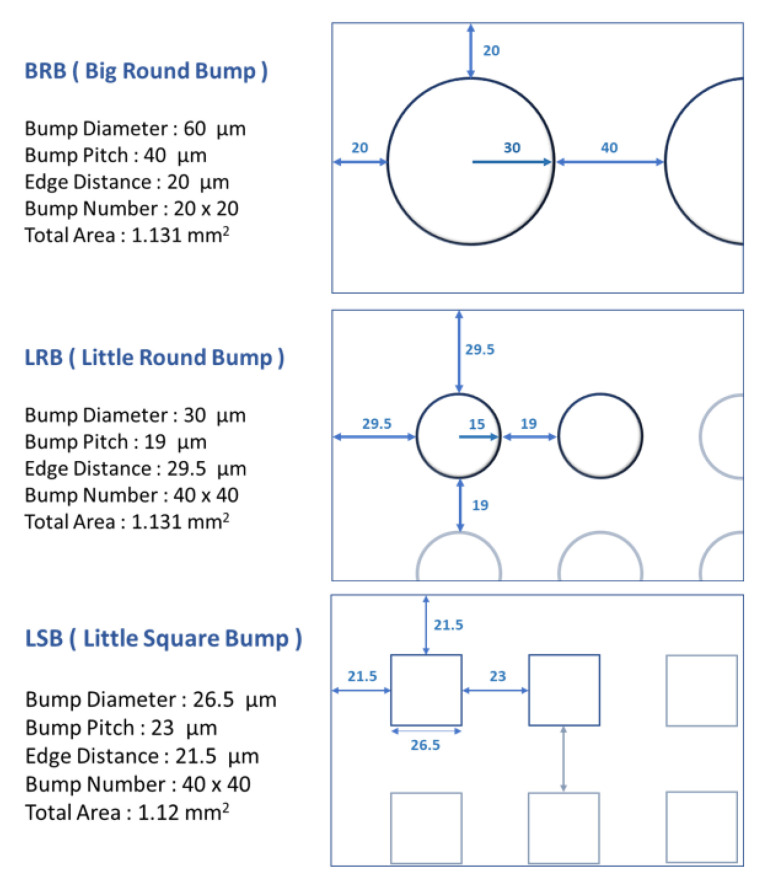
Specifications of the three bump types.

**Figure 4:**
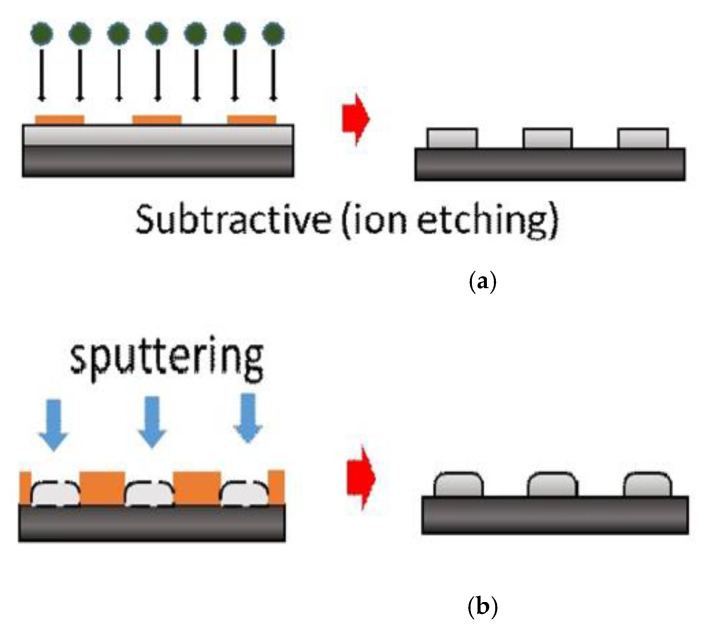
Illustrations of Al bump fabrication: (**a**) subtractive method and (**b**) additive method.

Figure 3 presents the photomasks designed for the BRB, LRB, and LSB. The chip size was 2  ×  2 mm^2^, and the total contact area of the bumps was kept constant at 1.12–1.13 mm^2^. Lithography and sputtering fabrication techniques were used according to the sample design. Ti-coated Si chips were used following subtractive lithography etching and additive lithography sputtering to create the three types of bump array. Figure 4a shows the subtractive method sequence to form Al bumps, in which the chip was sputtered with an Al film, followed by photo resist coating and lithography. Ion etching was applied to form Al bumps. Figure 4b displays the additive sequence, in which lithography was performed to etch out the defined patterns on the coated photoresist. Subsequently, sputtering was performed to deposit Al bumps, and after that the photoresist was removed.

### 2.3. Surface Pretreatment and Surface Energy Measurement

Surface modification of the Al bump surface was performed using Ar plasma prior to bonding. The conditions of Ar plasma were as follows: power of 200 W, gas flow of 50 sccm, and treating time of 20 min.

Surface energy was estimated according to contact angle measurement. The surface energy *γ_s_* can be divided into two parts, dispersive part $\gamma_{s}{}^{D}\;$ and polar part $\gamma_{s}{}^{p}$. Deionized water ($\gamma_{l}{}^{P}$ = 46.8 mN/m and $\gamma_{l}{}^{D}$ = 26 mN/m) and CH_2_I_2_ ($\gamma_{l}{}^{P}$ = 6.7 mN/m, $\gamma_{l}{}^{D}$ = 44.1 mN/m) were adopted to calculate *γ_s_* using Equations (1) and (2) [21].

(1) $$\gamma_{sl}=\gamma_{s}-\gamma_{l}\cos\theta$$

(2) $$\gamma_{sl}=\gamma_{s}+\gamma_{l}-2\left[{\left(\gamma_{s}^{D}+\gamma_{l}^{D}\right)}^{\frac{1}{2}}+{\left(\gamma_{s}^{P}+\gamma_{l}^{P}\right)}^{\frac{1}{2}}\right]$$

### 2.4. Ultrasonic Bonding and Joint Strength Measurement

**Figure 5:**
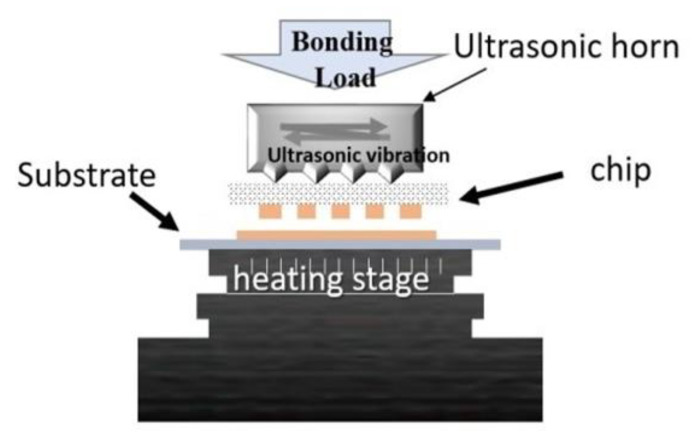
Schematic of the ultrasonic bonding machine.

The substrate specimens were square Al-coated Si chips with a length of 3 mm. The substrate was bonded with a Si chip with Al bumps on it through ultrasonic bonding. Figure 5 is a schematic of the customized ultrasonic bonding machine used in this study. The vibration frequency and amplitude of ultrasonic bonding were 35 kHz and 6 μm, respectively. The bonding process was performed in air or nitrogen atmosphere. The bonding temperature was 200 °C, the bonding load was 200 N, and the ultrasonic vibration time was 5 s.

**Figure 6:**
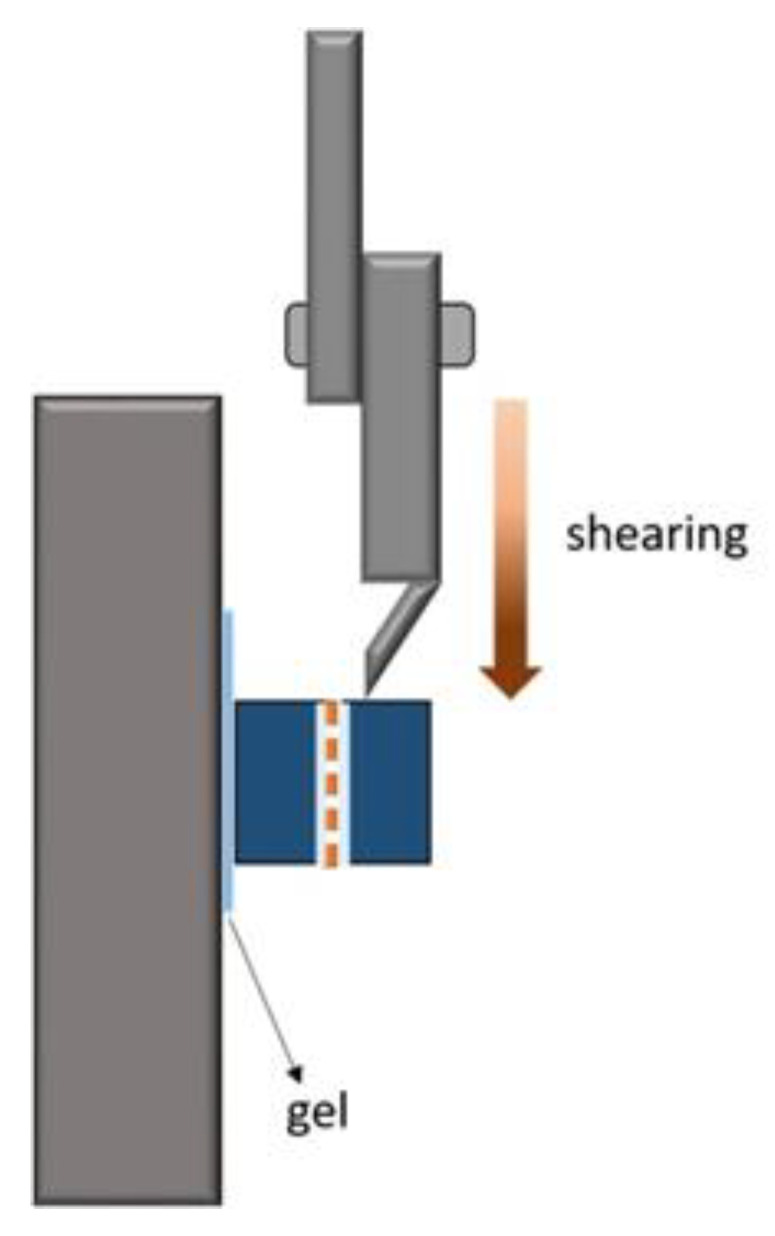
Illustration of the shear testing.

Figure 6 illustrates the shear testing for obtaining the joint strength. The shear rate was 0.2 mm/min. A thermosetting glue was used to fix the joint specimen, followed by pushing in the knife shear to adjust the shear plane for the shear test.

## 3. Results and Discussion

### 3.1. Simulation of Stress Distribution

**Figure 7:**
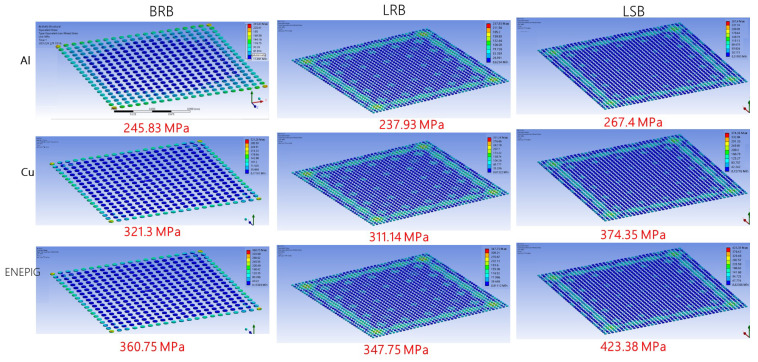
Stress distributions at the bonding interface when bump thickness was 10 μm.

**Figure 8:**
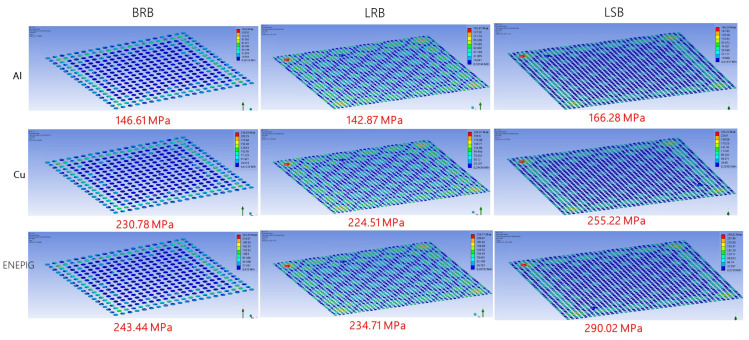
Stress distributions at the bonding interface when bump thickness was 5 μm.

**Figure 9:**
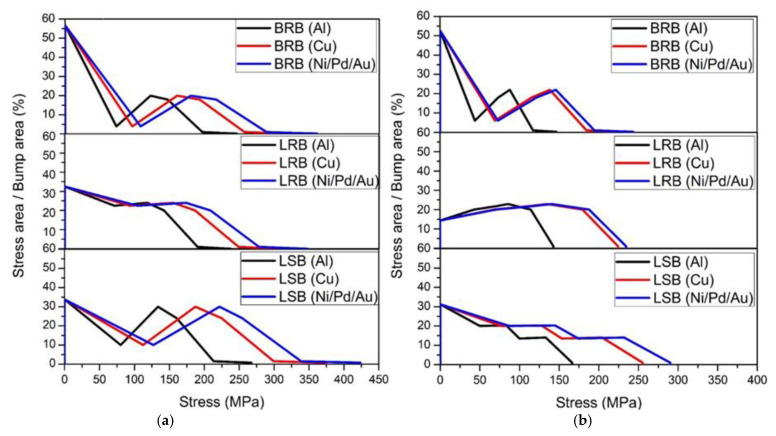
Quantitative simulated stress distribution on bonding surface and corresponding area fraction: (**a**) 10 μm bumps and (**b**) 5 μm bumps.

FEA was performed to simulate the stress distribution during ultrasonic bonding. Figure 7 and Figure 8, respectively, present the simulated stress distributions when the bump height was 10 and 5 μm. The maximum stresses were also labeled. The three bump types exhibited similar stress distributions, with the compressive stress concentrated on the edges of the specimens. Particularly, the BRB specimen exhibited relatively lower stress at the center, whereas the stress distributions of the LRB and LSB specimens were more even. For each material, the LSB exhibited a greater maximum compressive stress than the other two bump types, while LRB and BRB did not differ greatly. In addition, the 10 μm-thick bumps showed higher compressive stresses than the 5 μm-thick bumps for all the bump types. The statistics for the stress and corresponding area fraction illustrated in Figure 9 support the above observation about the stress distribution. Overall, stress was concentrated at the peripheral areas of the chips, and the bump shape influenced the stress distribution more than the bump size.

### 3.2. Simulated MAXIMUM STRESS with Respect to Materials and Bump Geometry

**Figure 10:**
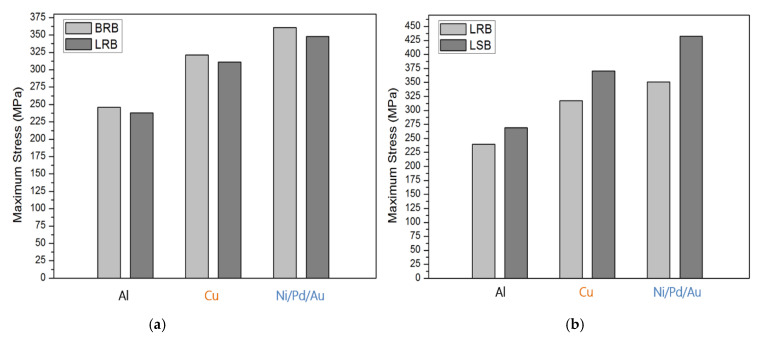
Comparison of the maximum stress of 10-μm thick specimens: (**a**) BRB and LRB; (**b**) LRB and LSB.

**Figure 11:**
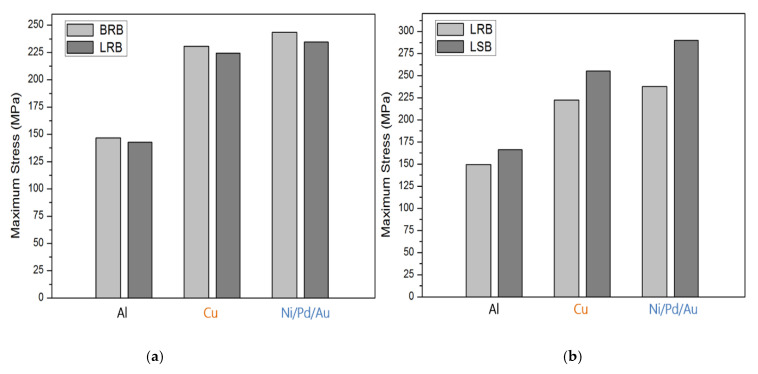
Comparison of the maximum stress of 5-μm thick specimens: (**a**) BRB and LRB; (**b**) LRB and LSB.

Figure 10 and Figure 11 compile the maximum stress determined in the simulation. For the 10 μm-thick bumps (Figure 10), the maximum stress comparisons all reveal that Ni/Pd/Au exhibited the largest stress, followed by Cu and Al. The maximum stress of the BRB was only slightly higher than that of the LRB for all materials, by approximately 5%; presenting little difference caused by size dissimilarity (Figure 10a). In contrast, the maximum stress of the LSB was approximately 15–20% greater than that of the LRB (Figure 10b). Figure 11 presents the simulation results when the bump height was 5 μm. The maximum thickness of the BRB was approximately 5% greater than that of the LRB, and that of the LSB was 10–20% greater than that of the LRB. The maximum stress of the 10-μm thick specimens was greater than that of the 5-μm thick specimens, and the increase in maximum stress depended on material type. Specifically, the Al specimens exhibited the largest increase (60%), followed by Ni/Pd/Au (45%) and Cu (40%).

### 3.3. Experimental Demonstration of Al/Al Joint Strength

**Figure 12:**
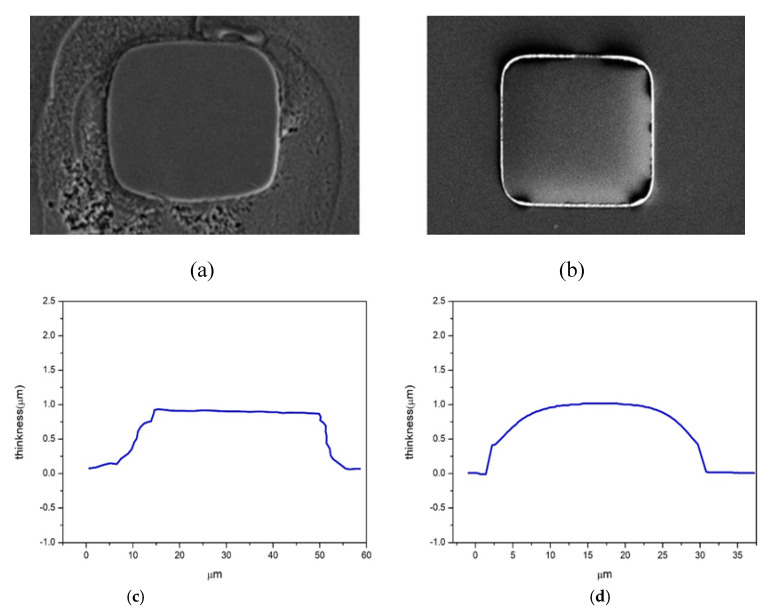
LSB: (**a**) top-down view of the subtractive Al bump; (**b**) top-down view of the additive Al bump; and cross-sectional atomic force microscopy scans of the (**c**) subtractive Al bump and (**d**) additive Al bump.

**Figure 13:**
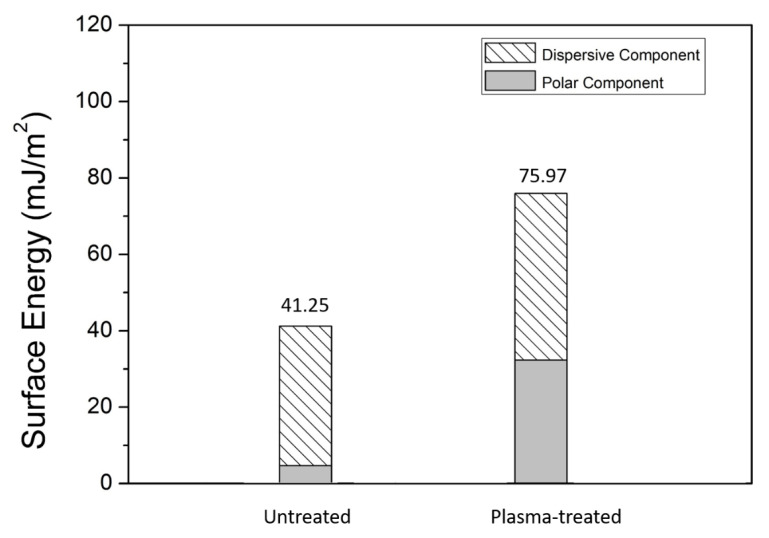
Surface energy of untreated and Ar-plasma treated Al surfaces.

Figure 12a,b present the top views of LSB Al bumps prepared using the subtractive and additive methods, respectively. The contours of the Al bumps shown in Figure 12c,d suggest than the bump height of these two samples was about 1 μm. The additive bumps exhibited an arched surface. On the contrary, the subtractive bumps showed a flat surface, with an effective contact surface area at least 40% larger than that of the additive bumps. The arched surface of additive bumps might have resulted from the shadowing effect of sputtering due to the photoresists. The data given in Table 2 indicate that when ultrasonic bonding was performed in air without surface pre-treatment, the subtractive LSB exhibited an average shear strength of 22.5 MPa, whereas the bonding of the additive LSB was unsuccessful. When ultrasonic bonding was performed in a nitrogen atmosphere, the average shear strength of the subtractive LSB increased slightly to 23.7 MPa, whereas the bonding of the additive LSB still failed. When surface activation right before bonding was performed using Ar plasma, the bonding of the additive LSB remained unsuccessful, but the shear strength of the subtractive LSB increased considerably to 44.6 MPa. The enhancement by Ar plasma bombardment could be ascribed to the significant increase in surface energy, indicating sufficient surface activation [22,23]. The significant increase in surface energy could be verified by the data shown in Figure 13, which were derived from the contact angles of untreated surface using Equations (1) and (2), which were 74.9° for H_2_O and 45.9° for CH_2_I_2_, while those of the Ar plasma-bombarded surface were 4.6° for H_2_O and 31.5° for CH_2_I_2_.

### 3.4. Geometrical Effects on Joint Strength

**Figure 14:**
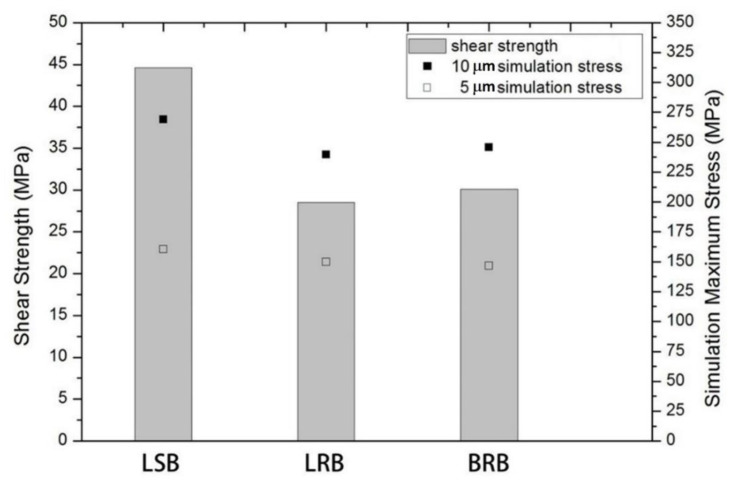
Simulated maximum stress and actual joint shear strength of the LSB, LRB, and BRB.

Figure 14 compiles actual joint strength and the maximum stress simulated using the aforesaid FEA on Al/Al bonding. When the bump thickness was 10 μm, the maximum stress of the LSB, LRB, and BRB was 267.4, 237.93, and 245.8 MPa, respectively. When the bump thickness was 5 μm, the maximum stress of the LSB, LRB, and BRB was 166.28, 142.87, and 146.61 MPa, respectively. As tabulated in Table 3, and also Figure 14, the shear test results reveal that the actual shear strength of the LSB, LRB, and BRB with surface pre-treatment was 44.6, 28.5, and 30.1 MPa, respectively, whose sequence approximated the maximum stresses estimated through simulation. This again indicates that under the same contact area, bump shape exerted a more notable effect on ultrasonic bonding than bump size. The test results for both the actual joint strength and the simulated maximum stress supported this inference.

Diffusion under pressure and stress has been studied thermodynamically [24]. The estimation of diffusivity under stress conditions is based on the following equation.

(3) $$\ln\left(\frac{D}{D_{0}}\right)=\left(\sigma -\sigma_{0}\right)\frac{V^{*}}{kT}$$

where *D* is the diffusivity under the set conditions, *D*_0_ denotes the diffusivity at as-received state, V* is atomic volume, and 0 respectively represents the stresses with or free from stresses. Accordingly, it can be inferred that an increased compressive stress brought about an increase in *D*/*D*_0_. Therefore, the enhanced self-diffusivity and thus accelerated atom diffusion resulting from the compressive stress accounts for the greater bonding strength of the square bumps.

## 4. Conclusions

In this study, ultrasonic bonding was utilized to perform direct metal bonding for bump arrays. FEA was adopted to explore the effects of bump shape, size, height, and material on the stress distribution on the bonding surface. An experimental demonstration of Al/Al joint strength was also carried out. The simulation results reveal that the stress distribution of the large bumps was more uneven than that of the small bumps, while the bump shape and height influenced the maximum stress more significantly. Material type did not exert a notable effect on stress distribution, but the maximum stress was positively proportional to the Young’s modulus. When ultrasonic bonding was applied to bond Al bumps and thin film, only the subtractive bump underwent successful bonding, and Ar plasma pretreatment magnified the actual joint strength by multiple folds: the actual joint strength of the subtractive LSB specimens with Ar plasma pretreatment reached 44.6 MPa. A comparison of the experimental and FEA results verifies that the tendency of the joint strength observed in the experimental demonstration was consistent with that of the maximum stress estimated using FEA.

## Figures and Tables

**Table 1:** Material property parameters in finite element analysis (FEA).

Bump	Al	Cu	Ni	Pd	Au
Density (g/cm^−3^)	2.7	8.96	8.908	12.023	19.3
Poisson’s Ratio	0.35	0.34	0.31	0.39	0.44
Young’s Modulus (GPa)	70	110	200	121	79
Coefficient of Friction	1.4	0.2	0.53	0.36	0.26

**Table 2:** Comparison of the ultrasonic bonding results of the LSB bumps created using subtractive lithography etching and additive lithography sputtering (the conditions of Ar plasma: 200 W−50 sccm−20 min).

Photolithography Type	Pre-Treatment	Bonding Environment	Joint Strength
Additive	Without pre-treatment	Air	Failed
		N_2_ atmosphere	Failed
	Ar plasma	N_2_ atmosphere	Failed
Subtractive	Without pre-treatment	Air	22.5 MPa
		N_2_ atmosphere	23.7 MPa
	Ar plasma	N_2_ atmosphere	44.6 MPa

**Table 3:** Actual joint strength of the BRB, LRB, and LSB subjected to Ar plasma pretreatment (200 W, 50 sccm, and 20 min) and ultrasonic bonding.

	Pre-Treatment	Bonding Environment	Joint Strength
**BRB**	Ar plasma	N_2_ atmosphere	30.1 MPa
**LRB**			28.5 MPa
**LSB**			44.6 MPa
